# The pacing strategy and technique of male cross-country skiers with different levels of performance during a 15-km classical race

**DOI:** 10.1371/journal.pone.0187111

**Published:** 2017-11-08

**Authors:** Boye Welde, Thomas L. Stöggl, Gunnar E. Mathisen, Matej Supej, Chiara Zoppirolli, Andreas K. Winther, Barbara Pellegrini, Hans-Christer Holmberg

**Affiliations:** 1 School of Sport Sciences, UiT The Arctic University of Norway, Tromsø, Norway; 2 Department of Sport Science and Kinesiology, University of Salzburg, Salzburg, Austria; 3 Swedish Winter Sports Research Centre, Department of Health Sciences, Mid Sweden University, Östersund, Sweden; 4 Faculty of Sport, University of Ljubljana, Ljubljana, Slovenia; 5 CeRiSM, Center of Research in Mountain Sport and Health, University of Verona, Rovereto, Italy; 6 Department of Neuroscience, Biomedicine and Movement Sciences, University of Verona, Verona, Italy; Universita degli Studi di Verona, ITALY

## Abstract

In this study the pacing strategy, cycle characteristics and choice of technique of elite male cross-country (XC) skiers during a three-lap, 15-km classical race with interval start were measured. During the Norwegian Championships in 2016, fast (n = 18, age: 26±4 yr; height: 182±4 cm; body mass: 78±3 kg (means±SD)) and slow skiers (n = 18, age: 22±2 yr; height: 183±5 cm; body mass: 78±6 kg) were video recorded on flat (0°), intermediate (3.5°) and uphill sections (7.1°) of the first and final laps. All skiers adopted a positive pacing strategy, skiing more slowly (11.8%) with shorter cycles (11.7%) on the final than first lap (both p<0.001; _p_η^2^ = 0.93 and 0.87, respectively). The fast skiers were 7.0% faster overall (p<0.001, *d* = 4.20), and 6.1% (p<0.001, *d* = 3.32) and 7.0% (p<0.001, *d* = 3.68) faster on the first and final laps, respectively, compared to slower skiers. On all sections of both laps, the fast skiers exhibited 9.5% more rapid (_p_η^2^ = 0.74) and 8.9% (_p_η^2^ = 0.48) longer cycles (both p<0.001). On intermediate terrain, the fast skiers employed primarily double poling (DP, 38.9% on the first lap) and double poling with a kick (DP_KICK_, 50% on the final lap). In contrast, the slow skiers utilized for the most part DP alone (lap 1: 33.3%, lap 3: 38.9%) or in combination with other techniques (lap 1: 33.3%, lap 3: 38.9%) and decreased their usage of DP_KICK_ from 27.8% on the first to 16.7% on the final lap. Skiing velocity on flat and intermediate terrain proved to be the best predictor of race performance (p<0.001). In conclusion, during a 15-km classical XC skiing race, velocity and cycle length decreased from the first to the final lap, most extensively on flat terrain and least uphill. Moreover, on the intermediate sections the fast and slow skiers chose to use different techniques.

## Introduction

During classical cross-country (XC) skiing races, the diagonal stride (DIA) and double poling (DP) techniques are most commonly employed on steep uphill and flat terrain, respectively [1, 2], while double poling with a kick (DP_KICK_) is utilized on moderate inclines [1–3]. Within the limits of their own individual physical and technical capacities, the skiers adapt their technique to the track profile and ski/snow friction [4–6].

During roller skiing on a treadmill at a fixed incline of 2°, Pellegrini et al. [1] found that all skiers chose DP as the speed increased. When the speed was fixed at 10 km/h, they all preferred to use DP at 0–1°, transitioning on steeper inclines first to DP_KICK_ (2–3°) and subsequently to DIA (>5°). These findings suggest that XC skiers cannot race successfully over longer distances across varying terrain utilizing DP alone.

However, exceptional improvements in ski equipment and snow-grooming techniques, as well as in the muscular strength and upper-body capacity of skiers over the past two decades, have led to substantial increases in the average speed of XC skiing competitions [5]. Moreover, the revolutionary rise in the use of DP, previously restricted primarily to level terrain, motivates detailed examination of the usage of the three major classical XC techniques by elite skiers during races. In this sport, the relative contributions of the arms and legs vary with the different techniques and course profiles [1, 5]. Thus, during a race upper- and lower-limb fatigue may also vary, thereby influencing selection of technique.

The ability to resist fatigue is, clearly, important for success in XC skiing and to this end various pacing strategies have evolved. During both sprint competitions and longer races, skiers generally employ a so-called positive pacing [7–9], i.e., after attaining peak speed, the athletes gradually slow down [10]. In connection with a 10-km skating race (two 5-km laps), Rundell and McCarthy [11] found that cycle velocity and length on the second lap demonstrated the most pronounced relationship to both lap time and total race time and, furthermore, that the slower velocity uphill during this lap was due to a reduction in cycle length, but not cycle rate. Similarly, Bilodeau et al. [8] observed that during a 50-km classical XC race consisting of 4 laps of equal length the mean velocity on the last two laps was slower than on the first two and that when employing DIA or DP on the flat and uphill (7°) sections, faster skiers demonstrated longer cycles, with no difference in cycle rate. Moreover, the slower skiers spent significantly more time on the uphill than flat sections [8].

Although courses for XC skiing races are designed to include approximately equal lengths of uphill, flat and downhill terrain, more than 50% of the total race-time is spent skiing uphill, and performance on these sections is thus regarded as the major determinant of success [4, 5]. Accordingly, Norman, Ounpuu, Fraser, and Mitchell [12] estimated that on uphill sections many skiers perform at an intensity greater than their maximal oxygen uptake and then utilize downhill sections for recovery. However, little is yet known about pacing behaviour on different slopes and its contribution to high-level performance in XC skiing has not yet been investigated thoroughly.

Even though several research groups have characterized the kinematics of classical XC cross-country skiing [1, 3, 8, 13–15], relatively little is presently known concerning the biomechanics of today’s elite skiers, as well as skiers with different levels of performance during an actual race, especially since the only reported analyses of distance races were performed as long ago as the 1980–90’s [6, 8, 12, 14]. In addition, these previous studies focused on flat and uphill sections, disregarding intermediate inclines.

Although these earlier studies have improved our understanding of the biomechanics of XC skiers with different levels of performance, they provide little information about choice of technique and its association with cycle characteristics and skiing velocity, as well as pacing strategies, during an actual classical competition. XC skiing has changed substantially in recent decades, e.g., through introduction of the skating style, new distances and mass starts. Such changes, together with technological innovations, better track preparations and developments in training, require research on today´s skiers with different levels of performance. Accordingly, the current investigation aimed to explore the skiing velocity, cycle characteristics and choice of skiing technique by world-class (fast) and national-level (slow) skiers on the flat, intermediate and uphill sections of the first and final of the three 5-km laps during the classical race at the Norwegian XC skiing championship for men in 2016. We hypothesized that (1) skiing velocity on the uphill sections is more closely related to overall performance than velocity on the flatter sections; (2) the cycles of faster skiers are longer and decline less in length than those of slower skiers; (3) faster skiers utilize DP to a greater extent on flat and intermediate terrain; and (4) usage of DP decreases from the first to the last lap.

## Materials and methods

### Subjects

**Fig 1:**
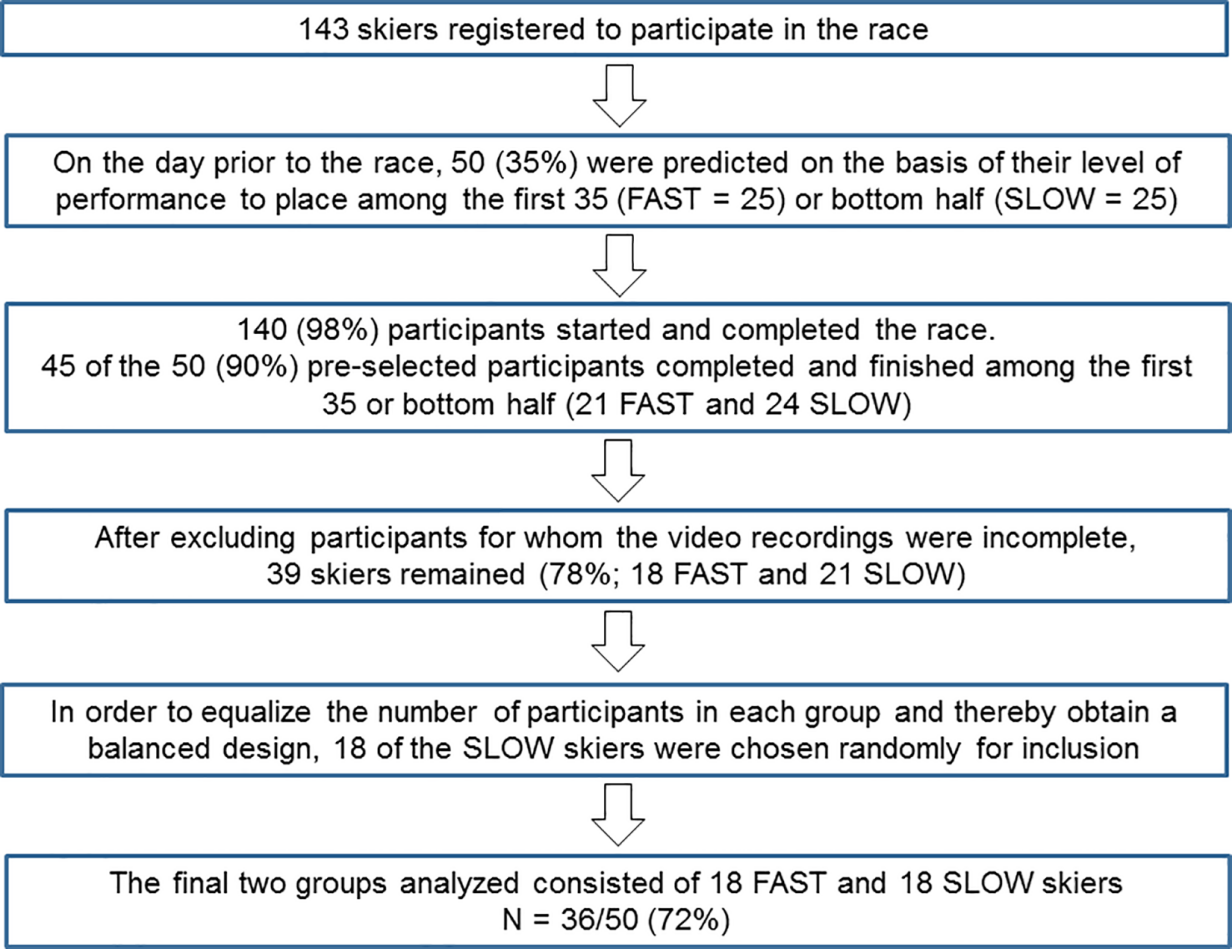
Flowchart illustrating our procedure for selection of participants.

Altogether, 36 male XC skiers selected on the basis of their performance in the 15-km classical race at the Norwegian XC skiing championships for men in Tromsø, 2016, volunteered to take part. Prior to the race, we selected 50 Norwegian skiers (foreign participants were excluded) for video recording– 25 predicted to be among the first 35 finishers (i.e., first-quartile, fast skiers) and 25 among the slowest half (slow skiers). Four skiers who did not fulfill these predictions were subsequently excluded and, moreover, three other fast skiers arrived at the site for video recording simultaneously, making it impossible to analyze at least three full cycles of movement. This left 18 fast skiers, including the winner of this race, three skiers ranked among the top 10 in the World Cup in 2016 and three medalists at World Championship or Olympic Games. To obtain two groups of equal size, we then randomly selected 18 of the slow skiers for analysis. Fig 1 illustrates this selection procedure.

The fast skiers finished on the average 121 ± 38 s (5.3 ± 1.7%, range: 2.3–7.6%) behind the winner, with the slow skiers 292 ± 39 s (12.9 ± 1.7%; range: 10.4–16.1%) behind him. The anthropometric characteristics and level of performance of our participants in distance races (FIS points) are presented in Table 1. This study, pre-approved by the Norwegian Social Science Data Services, was carried out in accordance with current ethical standards for sports and exercise research and all participants were informed of its nature before providing their verbal consent to participate and allow us to use their data.

**Table 1:** Anthropometrics and level of distance-race performance (expressed as FIS points) for 36 world- (fast) and national- (slow) class Norwegian cross-country skiers participating in the 15-km classical race at the Norwegian cross-country skiing championships for men in Tromsø, 2016.

Parameter	Fast skiers (n = 18)	Slow skiers (n = 18)	Cohen's *d*
Age (yrs)	26±4***	22±2	1.42
Body height (cm)	182±4	183±5	0.03
Body mass (kg)	77.6±3.2	77.7±5.7	0.19
FIS points	31±18***	130±24	4.49

All values presented are means ± SD.

***P < 0.001, in comparison to the corresponding value for the slow skiers.

### Procedures

**Fig 2:**
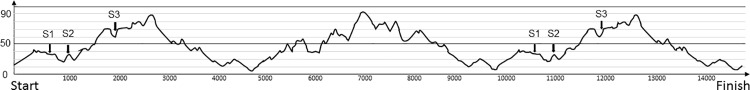
Profile of the 15-km classical cross-country skiing race course. The skiers covered three 5-km laps, two on track A (the first and final laps) and one on track B (the second lap). S1 (flat terrain, 0° incline), S2 (intermediate, 3.5°) and S3 (uphill, 7.1°) indicate the sections on which the skiers were filmed. See the text for further details.

The race involved two laps on ski track A and one on track B (A-B-A, see Fig 2), both 5-km long with approximately equal lengths of uphill, flat and downhill terrain and total climbs of 149 m and 185 m, maximal changes in elevation of 72 m and 76 m, and maximal climbs of 42 m and 38 m, respectively. The competitors were filmed on three different types of terrain during the first (after 0.8, 1.2 and 2.1 km) and final laps (after 10.8, 11.2 and 12.1 km) (Fig 2), with the flat (S1), intermediate (S2) and uphill sections (S3) having mean inclines of -0.3° (SD = 1.8), 3.5° (SD = 1.7) and 7.1° (SD = 0.7), respectively. The flat and intermediate sections were both 22 m in length and the uphill section 12 m.

On the flat section, the skiers were filmed in the middle of an approximately 100-m section, so that they were employing the DP technique before, during and after being recorded. On the intermediate section, recording was performed on a 22-m incline varying from 2–5°. The terrain immediately before and after this site was similar, i.e., 10 m sections each with an incline of ~ 2°. Thus, the total distance was approximately 40 m and the average slope 3°. The uphill section recorded was part of a longer climb with an inclination of 6–8° throughout.

Sony HDR-PJ810E video cameras (Sony corp., Tokyo, Japan) set at 50 Hz and shutter speed 1/500 s recorded the skiers in the sagittal plane at high resolution (1920 x 1080 progressive scan). These camcorders were positioned on top of tripods placed on custom-made wooden platforms, levelled with an electronic inclinometer 1 m above the ground and placed perpendicular to the track, at a distance of 12 m, 20 m and 25 m on the uphill, intermediate and flat sections. The ski tracks were in the centre of the field of vision and the camera focus and zoom set to cover at least three cycles of movement per section of terrain.

**Fig 3:**
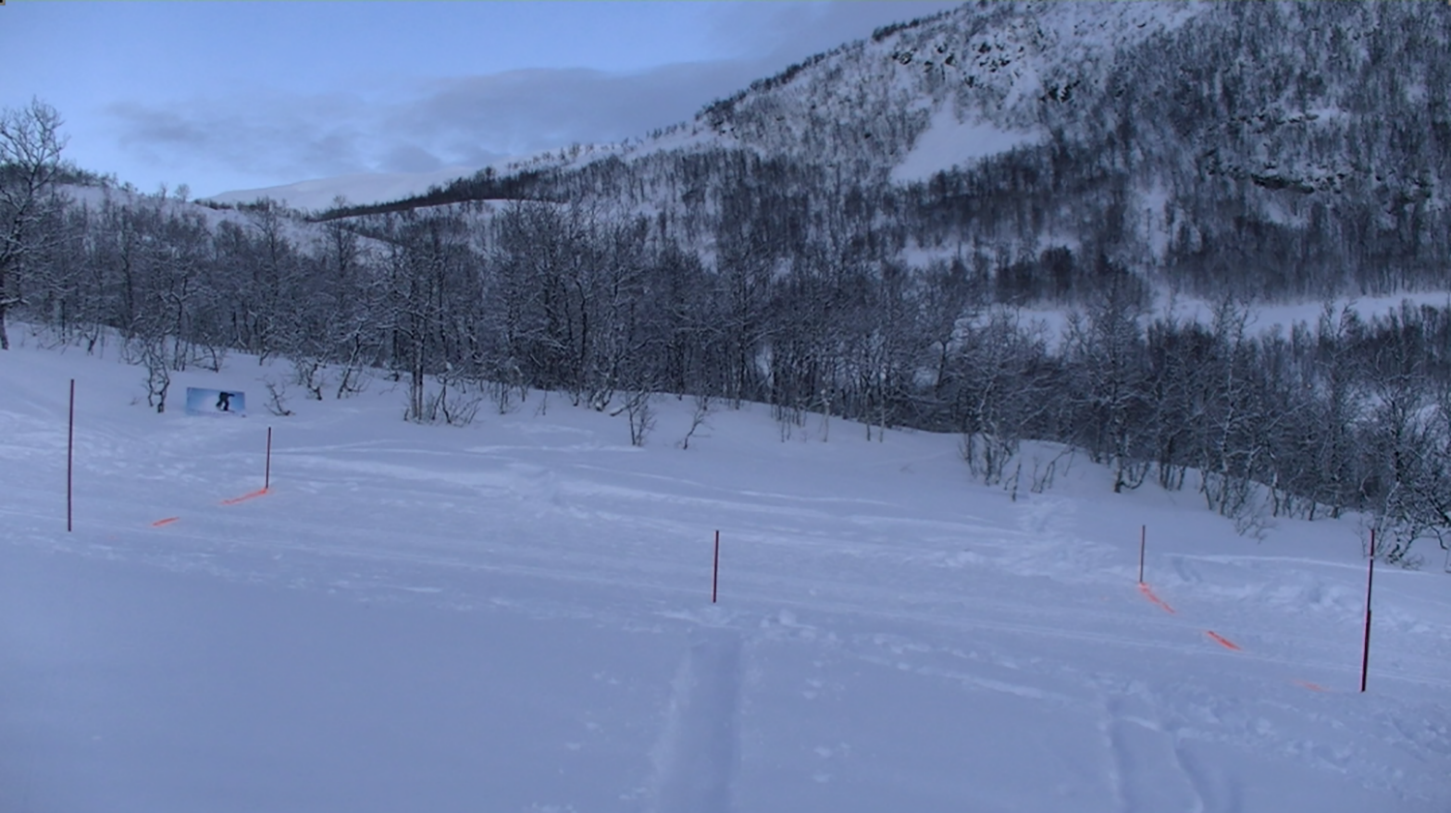
The sections video-recorded, showing the four red poles creating a perfect rectangle, the fifth pole set exactly in the middle of this rectangle, and the two orange lines drawn on the snow between the poles perpendicular to the ski track. This arrangement allowed appropriate calibration of the track chosen by each skier individually. This image was taken by the camera on the uphill section.

For purposes of calibration, prior to the race two red poles were placed at the beginning, one in the middle and two at the end of each section video-recorded. The two poles at the beginning and the two at the end formed a rectangle that enclosed the entire track and the fifth pole was placed exactly in the middle of the side of this rectangle closest to the camera (Fig 3). The distances between each pair of poles were determined with a measuring tape. In addition, two lines were drawn on the snow between the poles, perpendicular to the ski track, using orange fluorescent spray. This arrangement allowed us to calibrate our measurements for each individual track skied. No one skied outside the prepared tracks or changed track within the sections measured.

The weather during the race was stable, with no wind, air and snow temperatures of +1 and 0° C, respectively, and a relative humidity of 86%. The course was prepared with a grooming machine on the evening prior to the race and the team coaches considered the track conditions to be good, presenting no problems in choosing the optimal wax.

Prior to the race, each skier performed his own personal warm-up optimized for a classical 15-km race. All used their own racing poles and skis, selected for the prevailing snow conditions and waxed (grip wax: violet/universal klister) by experienced technicians.

### Data analysis and kinematics

**Fig 4:**
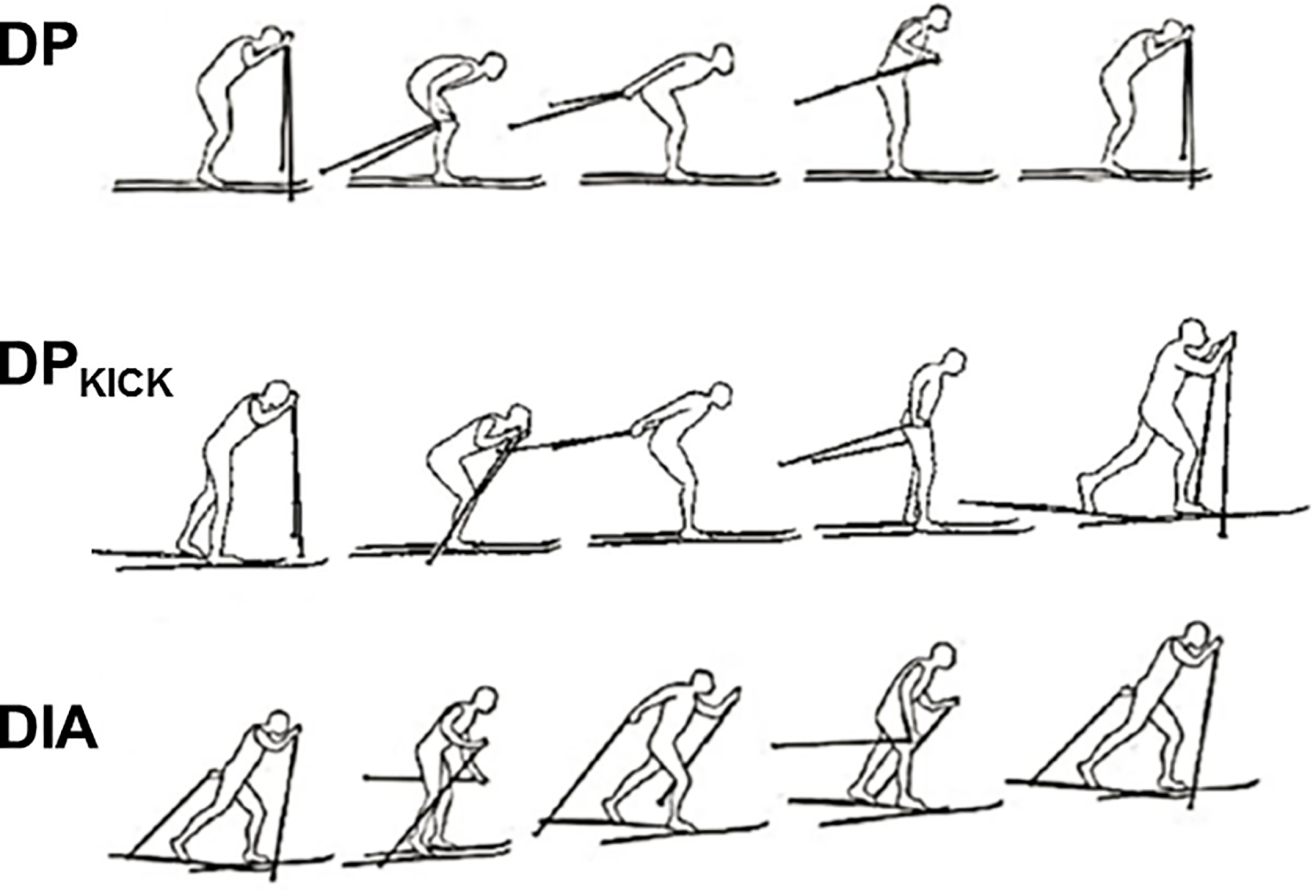
Overview of the techniques (gears) employed in classical cross-country skiing: DP (double poling), a symmetrical technique utilized on flat terrain, involves a pole push phase, which begins just before the skier plants his poles in the snow, and ends when the poles are lifted off the snow behind him. DP_KICK_ (double poling with a kick), employed on flat and slightly uphill terrain, involves a symmetrical poling action followed by a single step or kick with the left or right leg for propulsion. DIA (diagonal skiing), employed uphill, involves a kicking action followed by a weight shift to the gliding ski, after which the skier quickly performs a poling action with the arm opposite the kicking leg.

The performance and kinematics of the fast and slow skiers on the different sections were analysed. The total racing and 5 intermediate times for each skier were obtained from the race organizers, while performance (skiing velocity) within each section and cycle characteristics when employing the different techniques were determined from the video recordings. The techniques considered were DP, DP_KICK_ and DIA (Fig 4). In addition, on the intermediate section a fourth category MIXED was employed, since several skiers utilized a combination of two or even three of the techniques mentioned above. For all techniques, we defined the cycle as starting with the left pole plant and ending at the subsequent left pole plant. Thereby, DP_KICK_ cycles involved either one left- or right-leg kick.

Markers in the Kinovea software 8.25 [16] identified the start of a pole plant and the distance between two markers thus defined the cycle length. Cycle time was then taken as the period between the starts of two consecutive pole plants. Cycle rate was determined by dividing the camera speed by the number of frames occupied by one full cycle. Skiing velocity during each cycle was calculated as cycle length divided by cycle time. For purposes of analysis, we averaged the values for each parameter during at least three full cycles. When a skier utilized two or even three different techniques on the intermediate section, the parameters for these techniques were averaged.

### Statistical analysis

The data were confirmed to be normally distributed with the Shapiro–Wilk test and all results are presented as means ± *SD*. To compare skiing velocity and kinematic responses during the first and final laps, a 2 (first lap versus final) × 3 (sections 1–3) ANOVA with repeated measures and performance group (fast versus slow) was utilized. Post-hoc comparisons with Bonferroni corrections were conducted to detect differences. In those instances where the sphericity assumption was violated, *P***-**values were adjusted according to Greenhouse-Geisse**r**.

Multivariate ANOVA was applied to analyse the effects of level of performance and choice of technique (independent variables) on skiing velocity and kinematic variables (dependent variables) on the intermediate section of the first and final laps separately. Post-hoc comparisons with no adjustment for multiple comparisons were conducted to detect differences in choice of technique (DP, DP_KICK_, DIA, MIXED) by the fast and slow groups. The effect size for these ANOVA tests was evaluated as _p_η^2^ (partial eta squared) (with 0.01 < _p_η^2^ < 0.06 considered to be a small, 0.06 < _p_η^2^ < 0.14 a medium, and _p_η^2^ > 0.14 a large effect) or for the *t*-tests as Cohen´s *d* (0 < d < 0.2 considered to be a very small, 0.2 < d < 0.5 a small, 0.5 < d < 0.8 a medium and d > 0.8 a large effect) [17].

Associations between overall performance or kinematic responses and skiing velocity on each flat, intermediate and uphill section were estimated with the Pearson product-moment correlation coefficient, with interpretation according to Hopkins, Marshall, Batterham, and Hanin [18]: *r* < 0.1 = trivial, 0.1–0.3 = small, 0.3–0.5 = moderate, 0.5–0.7 = large, 0.7–0.9 = very large, 0.9–1.0 = nearly perfect, and 1.0 = perfect. Backward multiple linear regression was used to predict mean race velocity, i.e., race performance. The mean skiing velocity on the flat, intermediate and uphill sections served as predictors in this model. The level of statistical significance was set at *P* < 0.05. All statistical analyses were performed with the IBM SPSS Statistics for Windows, version 21.0 (IBM Corp., Armonk, NY, USA).

## Results

### Race performance and pacing strategy

**Fig 5:**
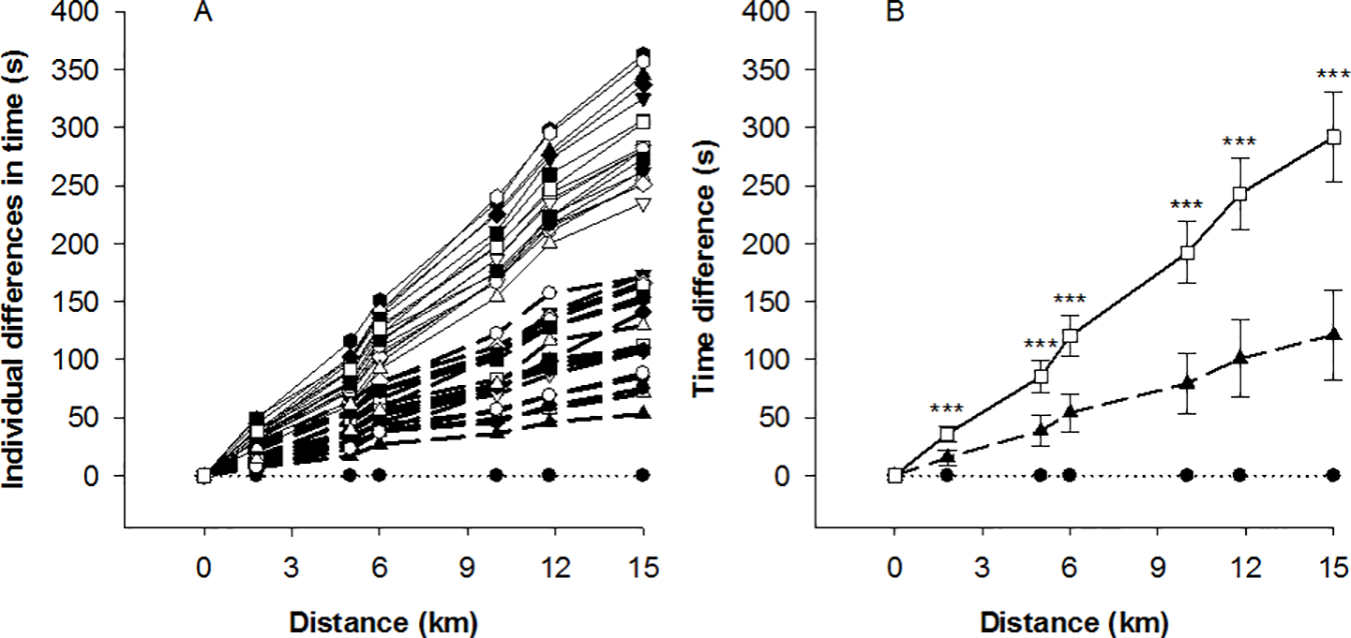
The time required to reach different points along the 15-km classical race course at the Norwegian cross-country skiing championship for men in Tromsø, 2016. (A) Individual slow (n = 18, solid line) and fast (n = 17, medium dash) skiers cluster in the upper and middle of the figure, respectively. The reference line at the bottom (dotted line, black circles) represents the winner. (B). Mean (SD) differences between the slow (n = 18, solid line, white squares) and fast skiers (n = 17, medium dash, black triangles). The reference line at the bottom (dotted line, black circles) represents the winner. *** p < 0.001, for comparison of the two groups.

The FIS distance points of each skier were closely associated with both his finishing time (r = 0.89 (very large), p < 0.001) and placement (r = 0.91 (nearly perfect), p < 0.001). The winner finished in 37 minutes and 47.9 seconds, skied without grip wax, used DP exclusively (unlike all other participants), and led from start to finish. The differences between the finishing time of the winner and the other individual skiers and between the two groups at different time-points along the course are illustrated in Fig 5. The time differential between the fast and slow groups increased from the first to the final lap (i.e., interaction lap x performance group; differential of 12 ± 26 s, p = 0.008, *d* = 0.91, large effect). In detail, the fast skiers were 7.0% (179 ± 86 s) more rapid overall (2381 ± 46 versus 2560 ± 39 s, p < 0.001, *d* = 4.20, large effect), 6.1% faster on the first lap (755 ± 16 versus 804 ± 13 s; p < 0.001, *d* = 3.32, large effect) and 7.0% faster on the final lap (796 ± 18 versus 857 ± 15 s; p < 0.001, *d* = 3.68, large effect).

All skiers demonstrated a positive pacing strategy (i.e., slowed down progressively after attaining peak speed) from the first to final lap, as well as on the flat section. In addition, all but one slow skier utilized positive pacing strategy on the intermediate section. In the case of the uphill section, 7 fast skiers and 11 slow skiers were faster (i.e., exhibited negative pacing, an increase in speed over the duration of a race) on the final lap; 10 and 7 were slower (positive pacing strategy); while one slow skier demonstrated even pacing.

### Skiing velocity

The skiing velocities and kinematic variables for all skiers on the different sections and laps are summarized in Table 2. The winner was fastest on the flat sections of both laps, second fastest on the intermediate terrain of both laps, and first and fourth on the uphill sections of the first and final laps, respectively.

**Table 2:** Skiing velocities and kinematic parameters for 36 world (fast) and national (slow) class Norwegian cross-country skiers on the flat (S1, 0° incline), intermediate (S2, 3.5°) and uphill sections (S3, 7.1°) of the 15-km classical race at the Norwegian cross-country skiing championships for men in Tromsø, 2016.

	Section 1, flat				Section 2, intermediate				Section 3, uphill			
*Technique*	DP				MIXED				DIA			
	Fast skiers (N = 18)		Slow skiers (N = 18)		Fast skiers (N = 18)		Slow skiers (N = 18)		Fast skiers (N = 18)		Slow skiers (N = 18)	
Parameter	First lap	Final lap	First lap	Final lap	First lap	Final lap	First lap	Final lap	First lap	Final lap	First lap	Final lap
Skiing velocity (m/s)	7.43±0.18***	6.04±0.20***^##^	6.88±0.29	5.56±0.19^###^	5.16±0.26***	4.63±0.24***^###^	4.61±0.19	4.20±0.31^###^	3.66±0.16***	3.61±0.35*	3.29±0.18	3.32±0.34
Cycle time (s)	1.08±0.06	1.11±0.08	1.09±0.05	1.13±0.07^##^	1.11±0.14	1.13±0.14	1.12±0.13	1.09±0.10	1.02±0.08	0.98±0.08^##^	1.03±0.04	0.99±0.10
Cycle length (m)	8.00±0.50**	6.69±0.53*^###^	7.52±0.40	6.28±0.49^###^	5.70±0.62*	5.21±0.56***^###^	5.15±0.62	4.58±0.41^###^	3.74±0.27***	3.53±0.36*^#^	3.39±0.22	3.27±0.32
Cycle rate (Hz)	0.93±0.06	0.91±0.06	0.92±0.04	0.88±0.05^#^	0.92±0.12	0.92±0.12	0.91±0.10	0.93±0.09	0.98±0.08	1.03±0.08^##^	0.97±0.04	1.02±0.09^#^

All values presented are means ± SD.

*P<0.05

**P<0.01

***P<0.001 significantly different from the corresponding value for the slow skiers.

^#^P<0.05

^##^P<0.01

^###^P<0.001 significant within-group change from the first to final lap.

DP = Double poling technique; DIA = Diagonal stride technique; MIXED = Mixture of techniques.

There was a significant main effect of level of performance on skiing velocity within the individual sections (F_1,34_ = 98.51, p < 0.001, _p_η^2^ = 0.74, large effect) and the fast skiers were 9.5% faster overall than the slow skiers. In detail, the fast skiers were 9.9% and 9.2% faster on the first and final lap and 8.3%, 11.1% and 9.9% faster on the flat, intermediate and uphill terrains, respectively (all p < 0.001, _p_η^2^ = 0.36–0.79, large effect). Moreover, the fast skiers were 8.0% (_p_η^2^ = 0.57, large effect) and 8.6% (_p_η^2^ = 0.62, large effect) faster on the flat sections of the first and final lap, 11.9% (_p_η^2^ = 0.60, large effect) and 10.3% (_p_η^2^ = 0.40, large effect) faster on the intermediate sections and 11.1% (_p_η^2^ = 0.55, large effect) (all p < 0.001) and 8.6% (_p_η^2^ = 0.16, large effect) faster on the uphill sections (p = 0.02). The interactions of level of performance x lap (p = 0.136, _p_η^2^ = 0.06, small effect) and level of performance x sections (p = 0.078, _p_η^2^ = 0.08, medium effect) were not statistically significant.

**Fig 6:**
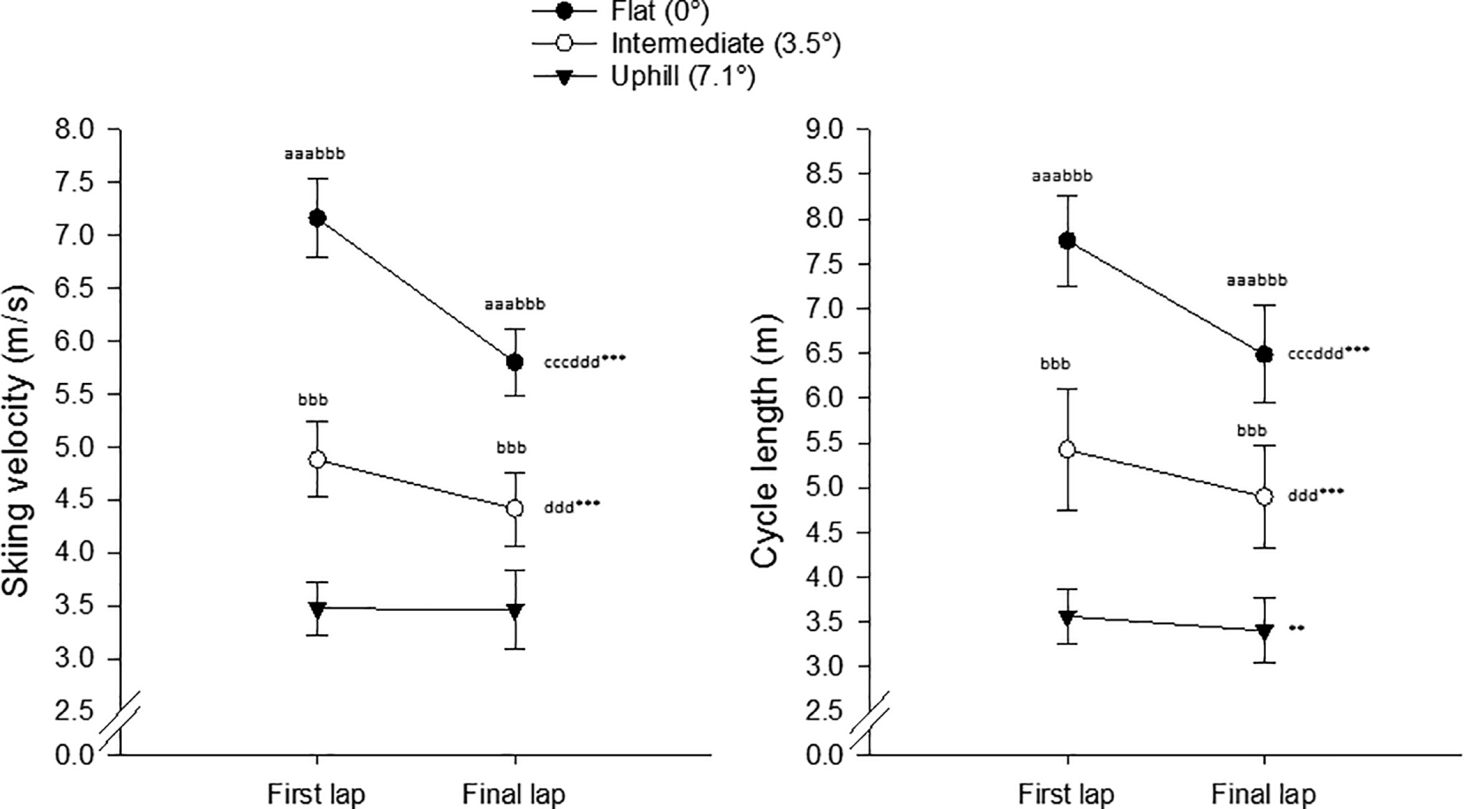
Skiing velocity (A) and cycle length (B) on the flat (0°), intermediate (3.5°) and uphill (7.1°) sections of the first and final laps during the 15-km classical race at the Norwegian cross-country skiing championship for men in Tromsø, 2016 (n = 36, mean ± SD). ^a^ Significantly different from the intermediate section, ^aaa^ P < 0.001; ^b^ Significantly different from the uphill section, ^bbb^ P < 0.001; ^c^ Significantly different change than in the case of the intermediate section, ^c^ P < 0.001; ^d^ Significantly different change than in the case of the uphill section, ^d^ P < 0.001; * Significant change for this section from the first to final lap, ** P < 0.01, *** P < 0.001.

Skiing velocity was 11.8% slower on the final than first lap (F_1,34_ = 443.01, p < 0.001, _p_η^2^ = 0.93, large effect). There was also a significant main effect of section on skiing velocity (F_2,68_ = 2461.10, p < 0.001, _p_η^2^ = 0.99), with 39.5% (*d* = 9.59, large effect) and 86.8% (*d* = 15.49, large effect) higher velocity on the flat than intermediate and uphill sections, respectively, and 34.0% (*d* = 5.39, large effect) higher velocity on the intermediate than uphill section (all p < 0.001). The skiing velocity declined to the greatest extent on the flat section (23.4%, *d* = 3.98, large effect) and less so on intermediate terrain (10.6%, *d* = 1.32, large effect) (both p < 0.001), with no change uphill (-0.3%, p = 0.84, *d* = 0.03, small effect) (interaction effect between sections and laps F_2,68_ = 201.44, p < 0.001, _p_η^2^ = 0.86, large effect) (Fig 6A).

### Cycle characteristics

The within-and between-group changes in all kinematic variables examined are shown in Table 2. The fast skiers employed 8.9% longer cycles than the slow skiers (F_1,34_ = 31.39, p < 0.001, _p_η^2^ = 0.48, large effect), with differences of 6.4% (p = 0.003, _p_η^2^ = 0.231, large effect) and 6.6% (p = 0.02, _p_η^2^ = 0.150, large effect) on the flat section of the first and final lap, respectively, 10.7% (p = 0.01, _p_η^2^ = 0.172, large effect) and 13.8% (p < 0.001, _p_η^2^ = 0.309, large effect) on the intermediate terrain and 10.2% (p < 0.001, _p_η^2^ = 0.342, large effect) and 8.1% (p = 0.03, _p_η^2^ = 0.138, medium effect) uphill. No significant interaction between level of performance x lap (p = 0.82, _p_η^2^ = 0.002, small effect) and level of performance x section (p = 0.30, _p_η^2^ = 0.04, small effect) was observed.

Furthermore, the cycles were 11.7% shorter on the final than first lap (F_1,34_ = 224.18, p < 0.001, _p_η^2^ = 0.87, large effect). The main effect of section (F_2,68_ = 794.91, p < 0.001, _p_η^2^ = 0.96, large effect) revealed that the cycle length declined with rising incline, decreasing on the uphill and intermediate terrain to approximately 50% (*d* = 10.85, large effect) and 72.5% (*d* = 4.33, large effect), respectively, of the cycle length on the flat section (both p < 0.001), with 48.2% longer cycles on intermediate than uphill terrain (p < 0.001, *d* = 4.25, large effect). From lap 1 to lap 3 the cycle length was reduced 19.6% on the flat (p<0.001, _p_η^2^ = 0.86, large effect), 10.8% on the intermediate (p < 0.001, _p_η^2^ = 0.54, large effect) and 4.8% on uphill (p = 0.004, _p_η^2^ = 0.22, large effect) terrain (lap x section interaction F_2,68_ = 54.16, p < 0.001, _p_η^2^ = 0.61, large effect) (Fig 6B).

Cycle time was similar for both groups (F_1,34_ = 0.06, p = 0.80, _p_η^2^ = 0.002, small effect) and laps (F_1,34_ = 0.10, p = 0.76, _p_η^2^ = 0.003, small effect). This time was 9.6% and 10.3% longer on the flat (*d* = 1.61, large effect) and intermediate (*d* = 1.12, large effect) than on the uphill section (both p < 0.001), with no difference between the former two (p = 1.00, *d* = 0.08, small effect) (main effect of section F_2,68_ = 21.56, p < 0.001, _p_η^2^ = 0.39, large effect). From the first to the final lap cycle time rose by 3.4% on the flat (p = 0.002, _p_η^2^ = 0.25, large effect), decreased by 4.0% on uphill (p = 0.007, _p_η^2^ = 0.19, large effect) and was unchanged on intermediate terrain (-0.5%, p = 0.69, _p_η^2^ = 0.01, small effect) (lap x section interaction F_2,68_ = 8.78, p < 0.001, _p_η^2^ = 0.21, large effect).

### Choice of technique

With the single exception of the winner, who employed DP exclusively during the entire race, all of the other skiers employed DP on the flat sections only and DIA only uphill. In contrast, as shown in Table 3, the selection of techniques on the intermediate section varied more widely, including DP, DP_KICK_, DIA, or a combination (MIXED) of two or even all three of these techniques. In short, the fast skiers employed DP to a greater extent on the first lap and more DP_KICK_ on the final lap than the slow skiers (Table 3).

**Table 3:** Relative usage of techniques (%), skiing velocity and kinematic variables for 36 world (fast) and national (slow) class Norwegian cross-country skiers on the intermediate section (S2, 3.5° incline) of the 15-km classical race at the Norwegian cross-country skiing championships for men in Tromsø, 2016.

		First lap					Final lap				
		DP	DP_KICK_	DIA^a^	MIXED	Mean	DP	DP_KICK_	DIA^a^	MIXED	Mean
Parameter	Group of skiers										
Techniques (%)	Fast (n = 18)	38.9	33.3	0	27.8		22.2	50.0	0	27.8	
	Slow (n = 18)	33.3	27.8	5.6	33.3		38.9	16.7	5.6	38.9	
	Mean (N = 36)	36.1	30.6	2.8	30.6		30.6	33.3	2.8	33.3	
	Fast (n = 18)	5.31±0.30***	5.05±0.26**		5.06±0.07***	5.16±0.26***	4.81±0.11*	4.53±0.22		4.66±0.30***	4.63±0.24***
Skiing velocity (m/s)	Slow (n = 18)	4.67±0.07	4.64±0.18	4.18	4.59±0.22	4.61±0.19	4.37±0.34	4.30±0.31	3.92	4.02±0.18	4.20±0.31
	Mean (N = 36)	5.02±0.40^##^	4.86±0.31^#^	4.18	4.80±0.29^#^	4.88±0.36	4.53±0.35	4.47±0.25	3.92	4.29±0.40	4.41±0.35
	Fast (n = 18)	0.96±0.06	1.26±0.06		1.13±0.05	1.11±0.14	0.99±0.07	1.23±0.08		1.04±0.09	1.13±0.14
Cycle time (s)	Slow (n = 18)	0.99±0.05	1.28±0.05	1.09	1.12±0.07*	1.12±0.13	1.00±0.08	1.20±0.03	1.06	1.13±0.08	1.09±0.10
	Mean (N = 36)	0.98±0.06	1.27±0.05^+++^^#^^$ $ $^	1.09	1.12±0.06^+++^	1.11±0.13	1.00±0.07	1.23±0.07^+++^^$ $^	1.06	1.09±0.09	1.11±0.12
	Fast (n = 18)	5.14±0.45*	6.34±0.24		5.71±0.30	5.70±0.62*	4.79±0.44	5.60±0.42		4.85±0.37	5.21±0.56***
Cycle length (m)	Slow (n = 18)	4.63±0.25	5.96±0.37	4.53	5.09±0.30	5.15±0.62	4.37±0.24	5.15±0.28	4.19	4.59±0.38	4.58±0.41
	Mean (N = 36)	4.91±0.44	6.17±0.35^+++^^###^^$ $ $^	4.53	5.37±0.43^++^	5.42±0.67	4.53±0.37	5.49±0.43^+++^^#^^$ $^	4.19	4.70±0.39	4.90±0.58
	Fast (n = 18)	1.04±0.06	0.80±0.03		0.89±0.04	0.92±0.12	1.01±0.08	0.82±0.05		1.02±0.10	0.92±0.12
Cycle rate (Hz)	Slow (n = 18)	1.02±0.05	0.78±0.03	0.92	0.91±0.06	0.91±0.10	1.00±0.08	0.84±0.02	0.94	0.91±0.06	0.93±0.09
	Mean (N = 36)	1.03±0.06^$ $ $^^¤¤¤^	0.79±0.03	0.92	0.90±0.05^¤¤¤^	0.91±0.11	1.01±0.07^¤¤¤^	0.82±0.05	0.94	0.95±0.10^¤¤¤^	0.92±0.11

^a^ Only one skier in the slow group applied DIA.

Skiing velocity and kinematics are presented as means ± SD.

*P<0.05

**P<0.01

***P<0.001 significantly different from the corresponding value for the slow group.

^#^P<0.05

^##^P<0.01

^###^P<0.001 significantly higher than DIA.

^+^P<0.05

^++^P<0.01

^+++^P<0.001 significantly higher than DP.

^$ $^P<0.01

^$ $ $^P<0.001 significantly higher than MIXED.

^¤¤¤^P<0.001 significantly higher than DP_KICK_.

DP = Double poling technique; DIA = Diagonal stride technique; MIXED = Mixture of techniques.

On the intermediate section, there was a significant main effect of level of performance on skiing velocity and cycle characteristics during both the first (Pillai´s Trace = 0.64, F_4,26_ = 11.72, p < 0.001; _p_η^2^ = 0.64, large effect) and final (Pillai´s Trace = 0.50, F_4,26_ = 6.44, p < 0.001; _p_η^2^ = 0.50, large effect) laps. Likewise, there was a significant main effect of choice of technique on these same variables during the first (Pillai´s Trace = 1.26, F_12,84_ = 5.04, p < 0.001; _p_η^2^ = 0.42, large effect) and final (Pillai´s Trace = 0.83, F_12,84_ = 2.69, p = 0.004; _p_η^2^ = 0.28, large effect) laps. There was no significant interaction between the level of performance and choice of techniques on either lap. All of these pairwise comparisons are documented in Table 3.

### Predictors of performance on the different sections

Cycle length was significantly correlated with skiing velocity on the flat, intermediate and uphill sections of both laps (0.35 ≤ r ≤ 0.74 (moderate to large effect); 0.001 ≤ p ≤ 0.01) whereas cycle rate (r = 0.40 (moderate effect); p<0.01) and cycle time (r = -0.40 (moderate effect); p < 0.01) were significantly correlated with skiing velocity only on the uphill section of the final lap.

The overall mean skiing velocity was significantly correlated to the mean skiing velocity on both the first (Flat: r = 0.82, Intermediate: r = 0.76, Uphill: r = 0.75; all large effects and all p ≤ 0.001) and final lap (Flat: r = 0.81; Intermediate: r = 0.72 (both large effects); Uphill: r = 0.47 (small effect); 0.001 ≤ p ≤ 0.01) and all of these velocities were significantly correlated to mean race velocity on the corresponding lap (0.46 ≤ r≤ 0.81 (small to large effect); 0.001 ≤ p ≤ 0.01).

Skiing velocity on the flat (ᵦ = 0.55, 95% CI: 0.39–0.70, p < 0.001) and intermediate (ᵦ = 0.24, 95% CI: 0.09–0.39, p = 0.002) terrain proved to be the best predictors of mean race velocity (F_2,33_ = 83.16, p < 0.001; R^2^ = 0.83, SEE = 0.10), while addition of skiing velocity uphill did not improve the prediction significantly.

## Discussion

The first major finding here is that all skiers employed positive pacing during the race, skiing more slowly on the final than the first lap. The greatest reduction in skiing velocity occurred on the flat section and the next most pronounced on the intermediate incline, with no reduction on the uphill terrain. The fast skiers were faster than the slow skiers on all sections of the first and final laps. Secondly, cycle length decreased as inclination increased, with the fast skiers employing longer cycles on all sections of the course. Furthermore, cycle length on all sections of the final lap was reduced in comparison to the first lap to the same extent for both groups, most pronouncedly on the flat terrain. In addition, on flat terrain, DP was the only technique used by both groups. Conversely, with the exception of the winner, who double-poled the entire race without grip-wax, DIA was the only technique employed uphill. However, on the intermediate sections the fast skiers chose DP and DP_KICK_ more extensively, whereas the slow skiers combined these two techniques and DIA to a greater extent. Thirdly, skiing velocity on all sections of both laps was strongly associated with both the corresponding lap velocity and mean velocity over the entire 15-km race. Skiing velocity on flat and intermediate terrain proved to be the best predictor of mean race velocity, with no improvement in predictive power when uphill velocity was included in the model. Finally, as mentioned above, the winner did not use grip wax and applied DP exclusively on all three sections of both laps.

### Race performance and pacing strategy

The winner led from start to finish and continuously placed more and more distance between himself and all other skiers. Furthermore, the slow skiers demonstrated larger variation in the time required to complete the first and, in particular, the final lap. The fast skiers were faster on all sections of both laps, as also observed by Bilodeau et al. [8] for a 50-km race. In our case, the fast skiers were approximately 11%, 10% and 8% faster on the intermediate, uphill and flat sections, respectively. The most likely explanation for this difference involves superior physical capacity uphill, better usage of DP and DP_KICK_ on flat terrain and more efficient adaptation of these techniques to the track profile.

The average age of our fast skiers was 26 (the winner was 32), while the corresponding value for the slow skiers was 22. Thus, these fast skiers may have developed their athletic performance [19], including factors of importance for endurance in skiing such as aerobic capacity, exercise economy, muscle strength and technical skills [20], to a greater degree. Such development probably allowed them not only to ski at high speed, but also to maintain a high pace throughout the race.

We do not know yet whether our present findings on male XC skiers are also relevant for women. The direct impact of upper-body capacity (endurance and strength) on the performance of XC skiers depends on the technique employed [21, 22], being, for example, greater with DP than DIA. Sex differences in choice of technique on different types of terrain and their potential relationship to overall performance remain to be investigated.

Here, all skiers spent more time on the final than the first lap, i.e., employed a positive pacing strategy. Such a strategy has also been observed in connection with sprint [7] and 15-50-km skiing races [8, 9], as well as in other sports [10]. Interestingly, with the exception of one slow skier, all of our skiers reduced their velocity on the flat and intermediate sections of the final lap; whereas 7 of the fast and 11 of the slow skiers were faster on the uphill section of the final lap. This observation indicates that an increase in ski drag from the first to final laps affected performance on the flat and intermediate sections, but had little influence when skiing up a steeper hill, where glide is only a minor factor. However, elite skiers may choose not to apply the same pacing strategy on all types of terrain and these skiers might have limited their velocity uphill to save energy for the flatter terrain. This observation differs from several earlier reports [7–9] and calls for further examination of pacing strategies during competitions in XC skiing, including the potential influence of ski drag, fatigue, track profile, weather and snow conditions, as well as waxing.

It should be mentioned here that in contrast to, e.g., running or cycling, frictional forces (in this case of skis on snow) can change considerably during a XC skiing race due, for example, to changes in temperature and exposure to sunlight, accumulation of dirt on skis (e.g., residues of klister from the skis of other skiers), and mechanical deterioration and other changes in the snow as many skiers pass over the track surface. Therefore, later in a ski race, substantially greater mechanical work might be required to maintain the same speed as earlier in the same race, which might be an additional explanation for why most skiers were slower on flat and intermediate sections of the last lap. Accordingly, it is possible that despite the clear reduction in speed from early to later in the race due to elevated drag forces, our skiers utilized an "internal" pacing strategy designed to maintain a constant metabolic rate, rather than "external" pacing.

This might also explain why the difference in steep uphill speed early and later in the race was relatively small. Uphill, the glide phase is short and the skiers work primarily against gravity, whereas on flat terrain they work against snow and air drag. However, for some as-yet-unknown reason the time difference between our fast and slow skiers was greater on the final than the first lap, which argues against any change in external forces, since this would be expected to affect fast and slow skiers equally.

### Kinematics and choice of skiing technique

Our fast skiers exhibited longer cycles on all sections of both laps with no alteration in cycle rate on the flat and the intermediate sections, and they were faster on the flat terrain. In contrast, Bilodeau et al. [8] found no difference in cycle length between faster and slower skiers on flat terrain. Furthermore, on the flat section of the final lap both of our groups shortened their cycle length and thereby lowered their skiing velocity, with the slow skiers also reducing their cycle rate.

On the uphill section as well, the higher velocity of our fast skiers was due to longer cycle length, rather than more rapid cycles. Similarly, Bilodeau et al. [8] reported that faster skiers utilized longer cycles on the first of four uphill sections during a classical 50-km race. In the present case, the cycle length of the fast skiers was shorter on the final than first lap, but this was compensated for by a faster cycle rate, so that their skiing velocity remained the same. The slow skiers also elevated their cycle rate on the uphill sections of the final lap, thereby maintaining velocity without shortening their cycles. However, the difference observed here in the cycle length of fast and slow skiers on uphill terrain indicates the importance of this aspect of performance on the outcome of distance races in XC skiing.

Both groups utilized DP exclusively on flat terrain and DIA exclusively uphill, in accordance with the findings of Pellegrini et al. [1] that DP is the technique preferred for XC skiing at high speed on flat terrain and DIA the preferred classical technique on steeper terrain (≥6°). However, our investigation appears to be the first assessment of kinematics on intermediate terrain during a real ski race. In contrast to the flat and uphill sections, on the intermediate incline of 3.5°, the use of DP, DP_KICK_ and DIA varied widely, with some skiers combining two or even three of these techniques. On the first lap, the fast skiers employed DP and DP_KICK_ 39% and 33% of the time, respectively, and DP_KICK_ alone or in combination with DP 50% and 28% of the time on the final lap. We propose that this shift in technique reflects the need to utilize DP_KICK_, which involves both the upper and lower body, to maintain skiing velocity.

In contrast, on both laps the slow skiers utilized for the most part DP alone (33%) or in combination with other techniques (39%), reducing their usage of DP_KICK_ from 28% on the first lap to 17% on the last. This distinct difference in choice of techniques may indicate that fast skiers coordinate their movements more efficiently and more effectively choose the appropriate technique for each type of terrain, a unique aspect of XC skiing performance [1, 23]. Moreover, the more extensive use of DP and DP_KICK_ by the fast skiers on the intermediate section may reflect differences in the strength-endurance capacities of the upper body. However, the slower speed of the other group may have influenced both their choice of techniques and when to change techniques, which could complicate generalization.

The fast skiers employed longer cycles than the slow skiers on the intermediate section of both laps, with no difference in cycle time or rate. More specifically, they were faster on this section on both laps when using DP alone or in combination with DP_KICK_ and/or DIA on both laps, but only faster on the first lap with DP_KICK_. This may reflect the fact that, in general the slower skiers reduced their use of DP_KICK_ on the final lap, while those who actually chose to continue using DP_KICK_ adapted more effectively to the track profile. Moreover, DP_KICK_ was associated with longer cycles and slower cycle rates than any of the other techniques on both laps, suggesting that the additional propulsive action of the legs provided by this technique may allow the skiers to better overcome the resistance of gravity and generate longer cycles, even on intermediate terrain.

### Predictors of performance

Cycle length was associated with mean skiing velocity on all three types of terrain on both laps, whereas more rapid cycles were only correlated to skiing velocity on uphill terrain in the final lap. This is partially in line with the conclusion by Bilodeau et al. [8] that cycle rate does not discriminate between faster and slower skiers. Altogether, increasing cycle length appears to play an important role in achieving high velocity during distance races in XC skiing.

The choice of technique for each combination of slope and speed should aim to optimize cycle rate while maintaining good cycle length. Our fast skiers appear to have achieved an effective balance between optimal cycle rate and distance travelled during each cycle by employing DP_KICK_ extensively on the intermediate section of the final lap. Indeed, in anticipation of fatigue on the final lap, the skiers may have decided to use a technique in which propulsion is aided by the legs in order to maintain a long cycle and thereby high speed.

Skiing velocity on the flat, intermediate and uphill sections of both laps correlated positively to the mean velocity on the corresponding lap, as well as the mean velocity over the entire race. Bolger et al. [9] also found significant correlations between overall performance and speed on uphill (r = 0.71) and flat (r = 0.77) terrain during a men´s classical race. However, although more than 50% of the total racing time is spent skiing uphill, in our present study mean race velocity was much more closely related to the velocities on flat and intermediate than on uphill terrain. Thus, the former two velocities, in contrast to the findings of Bolger et al. [9], proved to be the best predictors of mean velocity during this 15-km race, whereas addition of uphill velocity did not improve the prediction significantly.

Accordingly, performance on flatter terrain may be more important to the outcome of classical XC skiing than previously believed. Our observations may reflect the fact that, while reducing their velocity on the flatter terrain by 10–19%, the skiers maintained their velocity (± 1.4%) on the uphill sections. The present investigation appears to be the first use of a model involving different types of terrain (uphill, flat and downhill sections, also dividing the flat terrain defined by Bolger et al. [9] into flat and intermediate terrain) to predict overall performance during a classical XC skiing race.

Skiing without kick-wax and employing DP exclusively, the winner of the race finished 53 seconds before the second-place skier. He led from start to finish and was fastest on the flat section and second faster on the intermediate section of both laps. On the flat terrain, where all of the skiers employed DP, the winner had the longest cycles on both the first and final laps. More surprisingly, he was also first and fourth fastest on the uphill section (7.1°) of the first and final laps, respectively.

Earlier reports revealed that DP is the preferred XC skiing technique on inclines of up to 4° [1, 8, 24]. However, Stöggl and Holmberg [25] recently described how the strong development of modern elite skiers enables them to employ DP with good technique and high speed even at an incline of 7°. Thus, the winner of the classical 15-km race at the Norwegian XC skiing championship in Tromsø in 2016 demonstrated for the first time that by employing DP exclusively, it is possible not only to win on hilly terrain, but also to be among the fastest skiers uphill.

The enhanced usage of DP over the past few decades probably reflects more training of upper-body strength, as well as of this specific technique by elite skiers (e.g., endurance training of the upper body on roller skis, strength training in the gym and more specific training of DP power and speed) [5, 23]. Recently, in line with this investigation, Vandbakk et al. [26] found that inclusion of upper-body sprint-interval training improves maximal upper-body strength and VO_2max_ in female XC skiers to a greater extent than continuous endurance training. The development of equipment, including the poles, has also contributed to more extensive utilization of the DP technique [23].

### Strengths, limitations and practical applications

Major strengths here include the high level of skiing performance by and relatively large number of our participants, as well as our analysis on three different types of terrain during a real race on snow. In addition to being the Norwegian Championships, this race was important for qualification for participation in the World Championships four weeks later. Moreover, the structure of this race (laps A-B-A) allowed us to monitor the skiers on the same section of terrain at both early and late stages.

Data collection in an authentic outdoor setting involves both advantages and disadvantages. Here, we analyzed kinematics on three specific sections of terrain, but it would be valuable to also monitor the skiers over an entire course employing even more sophisticated methodology (for example, combining video recording, high-end real-time kinematic analysis with GNSS, and a full-body inertial motion caption suit). Unfortunately, this was not compatible with our aim to study a large number of skiers with minimal disturbance during this important race. At the same time, we decided against also using less advanced GNSS (e.g., a GPS wrist watch) to examine pacing strategy over the entire course, since although these devices do register speed accurately, their precision when measuring distance remains limited and they cannot monitor segment movement.

Video recording the skiers from one side with our high-definition cameras allowed us to monitor all of the body segments required for our analysis with an estimated error of less than 2–3 cm. However, video recording skiers during an interval start is challenging, since they occasionally arrive at the measuring station simultaneously and a few subjects had to be excluded. Changes in external conditions during the race (see also above) can potentially influence both the interpretation and generalization of the type of field measurements performed here. Although external conditions were stable throughout this 15-km race and, moreover, the effects of any alterations during such a short race should be minimal, we have no specific information on ski-snow friction, including possible changes during the race. Altogether, even though the course profile here conformed to FIS regulations, different results might be obtained on other types of courses and/or under other conditions.

The increased understanding of pacing strategy, the biomechanics and choices of the different classical skiing techniques on various types of terrain, as well as the relationship between a skier’s performance on sections of a race to his overall performance provided by our present findings has practical implications. Thus, skiers at all levels can improve their performance with more specific training of technique on different inclines, at different speeds and under varying conditions, in combination with training of endurance and, if necessary, more strength.

## Conclusions

Assuming the ski drag forces did not change, all of our skiers apparently employed positive pacing strategy during the race, with more rapid and longer cycles on the first than the final lap. At the same time, the difference in time between the fast and slow skiers was higher on the final than the first lap. The skiing velocity on the flat and intermediate sections was faster during the first than final lap, to the same extent for both groups, with no change in skiing velocity uphill. In addition, skiing velocity was slower and cycle length shorter during the final lap, especially on the flat section. The winner double-poled the entire race, while almost all of the remaining skiers utilized the same techniques on the flat and uphill sections; whereas on the intermediate terrain there was more variation between DP, DP_KICK_ and DIA, with some skiers combining two or even three of these techniques. Moreover, skiing velocity on all three types of terrain was significantly associated with the mean skiing velocity during both laps, whereas only the skiing velocity on the flat and intermediate sections significantly predicted mean race velocity, an observation that requires further examination. Furthermore, during this 15-km race the fast skiers employed DP and DP_KICK_ to a greater extent on intermediate terrain than the slow skiers. Accordingly, maintaining performance on the flat and intermediate sections, especially when utilizing the DP and DP_KICK_ techniques, might be particularly important in connection with distance XC skiing races.

## Supporting information

S1 File Supporting data. (XLSX)
